# Endophthalmitis caused by *Burkholderia cepacia* complex (BCC): clinical characteristics, antibiotic susceptibilities, and treatment outcomes

**DOI:** 10.1186/s12348-023-00370-1

**Published:** 2023-11-03

**Authors:** Flavius A. Beca, Jesse D. Sengillo, Hailey K. Robles-Holmes, Prashanth G. Iyer, Darlene Miller, Nicolas A. Yannuzzi, Harry W. Flynn

**Affiliations:** Department of Ophthalmology, Bascom Palmer Eye Institute, University of Miami Miller School of Medicine, Miami, FL USA

**Keywords:** Endophthalmitis, *Burkholderia cepacia*, Postoperative, Antibiotics, Enucleation

## Abstract

**Purpose:**

To report the clinical characteristics, antibiotic susceptibilities, and review the literature of *Burkholderia cepacia* complex (*BCC*) associated endophthalmitis.

**Study design:**

Retrospective, observational case series.

**Methods:**

Clinical and microbiology records were reviewed for patients evaluated at the Bascom Palmer Eye Institute and diagnosed wisth culture-confirmed endophthalmitis due to *BCC*. Antibiotic susceptibility profiles were generated using standard microbiologic protocols via an automated VITEK system.

**Results:**

Endophthalmitis associated with *BCC* was diagnosed in three patients. Infection occurred in the setting of post-penetrating keratoplasty (PKP), glaucoma filtering surgery, and suspected trauma. All isolates demonstrated in vitro susceptibility to ceftazidime and meropenem. Presenting visual acuity (VA) ranged from hand motion to light perception. Initial treatment strategies included intravitreal ceftazidime (2.25 mg/0.1 mL) and vancomycin (1.0 mg/0.1 mL) injections with fortified topical antibiotics in 2 patients, and surgical debridement of a corneoscleral melt with patch graft along with both topical fortified antibiotics oral antibiotics in the third patient. In all 3 patients, there was no VA improvement at last follow-up, as 2 eyes ultimately underwent enucleation and 1 eye exhibited phthisis bulbi at last follow-up. BCC related endophthalmitis was reviewed among 13 reports. Treatment outcomes were generally poor and antibiotic resistance was common. These BCC isolates cases demonstrated broad resistance patterns, with susceptibilities to ceftazidime (58%), ciprofloxacin (53%), and gentamicin (33%).

**Conclusions:**

Endophthalmitis caused by *B. cepacia* is a rare clinical entity with generally poor visual outcomes despite prompt treatment with appropriate antibiotics.

## Introduction

Bacterial endophthalmitis is most often caused by gram-positive organisms, but gram-negative organisms account for up to 30 percent of cases [24, 35]. Gram-negative endophthalmitis are often associated with unfavorable VA outcomes [25, 28]. The *Burkholderia cepacia* complex (BCC), previously classified as part of the *Pseudomonas* genus, is a group of at least 20 closely related species of rare gram-negative, oxidase-positive, non-fermenting bacillary organisms [10, 15].

Found in soil, water, plants, industrial settings, and hospitals, *BCC* is ubiquitous in the environment and harbors a large genome encoding for many virulence factors which provide natural antibiotic resistance [34, 36]. *BCC* has been implicated in outbreaks from contaminated surgical compounds, [12, 31] as well as pharmaceutical products such as nebulization solutions, hand sanitizers and mouthwash [1, 14, 32]. Tavares and colleagues reported viable BCC isolates from pharmaceutical-grade, nutrient-deficient, saline solutions containing biocidal preservatives as much as 16 months later in two BCC species [33].

Clinical and ecological distinction among the various BCC species remain to be described, and indeed, many clinical laboratories struggle with species specific identification using standard automated commercial systems [10, 13]. Cystic fibrosis, chronic granulomatous disease, and other causes of immune compromise have been cited as predisposing risk factors for *BCC* infections [21], although no such association has been reported yet for ocular infections.

Ocular *BCC* infections are rare and present with a significant degree of clinical variation. In a recent report of 6 cases of *B. contaminans* endophthalmitis following cataract surgery, time to onset of symptoms was 12–112 days, with 5 of 6 cases having at least one recurrence of symptoms following treatment [14]. Most case series of *BCC* endophthalmitis reported poor visual outcomes that often result in phthisis, enucleation or visual acuity of light perception [6, 11, 12, 16, 17, 20, 26, 31]. Deb et al*.* reported an outbreak of 5 cases of post-operative *BCC* endophthalmitis all of which failed to respond following vitreous tap and injection of vancomycin and ceftazidime, despite subsequent demonstration of ceftazidime susceptibility. 4 cases underwent vitrectomies with additional injection of meropenem, all achieving vision of 20/200 or better, of which 2 cases attained final vision of 20/60 [7]. In significant contrast [14]. reported all 6 cases having good visual outcomes of ≥ 0.8 [14]. Two other case reports of *BCC* endophthalmitis by Okonkwo [18] and Saffra [27] following anti-VEGF injections with relatively quick onset (4 days [18] and 11 days [27] following injection), treated with pars plana vitrectomy and intravitreal antibiotic injections also reported favorable visual outcomes (20/60 [18] and 20/30 [27] respectively). In a large review, *BCC* accounted for 1.8% (14/744) [26] of culture positive cases of endophthalmitis, which occurred after cataract surgery in 85% of cases (29/34), [16] and post-penetrating keratoplasty (PKP), LASIK, [23] intravitreal anti-VEGF injection, and filtering surgery in the remainder of cases.

Herein we report 3 cases of *B. cepacia* endophthalmitis evaluated and treated at our institution. We also review the literature of the other BCC endophthalmitis with particular attention to the reported antibiotic susceptibilities and clinical outcomes.

### Patients and methods

This retrospective consecutive case series conducted at Bascom Palmer Eye Institute examined the bacterial isolates of patients with culture-positive *B. cepacia* endophthalmitis (Table 1). Data collected from charts included patient demographics, preexisting ocular and systemic conditions, exam findings, treatment administered, operative reports, and microbiology laboratory results (Table 2). Phenotypic antibiotic resistance patterns reported here were obtained from minimum inhibitory concentration values using the automatic system Vitek 2 (bioMérieux, Durham, NC).

**Table 1:** Susceptibility testing results

	Case 1	Case 2	Case 3
Growth	Light	Heavy	Light
Ceftazidime	Susceptible	Susceptible	Susceptible
Meropenem	Susceptible	Susceptible	Susceptible
Trimethoprim + Sulfamethoxazole	Resistant	Susceptible	Resistant
Minocycline	Susceptible	Susceptible	NT
Amikacin	NT	NT	Susceptible
Cefepime	NT	NT	Susceptible
Ciprofloxacin	NT	NT	Susceptible
Gentamicin	NT	NT	Susceptible
Levofloxacin	NT	NT	Susceptible
Piperacillin + Tazobactam	NT	NT	Resistant
Tobramycin	NT	NT	Susceptible

*NT* Not tested, signifies antibiotic susceptibilities that were not tested

**Table 2:** Patient characteristics

Patient Characteristics	Case 1	Case 2	Case 3
Age	80 years old	66 years old	62 years old
Gender	Male	Female	Male
Baseline VA	HM	LP	N/A
Presenting VA	HM	LP	LP
Presenting Symptoms	Painful red eye, hypopyon	Painful red eye, hypopyon	Painful red eye, corneoscleral melt
Final VA	NLP	NLP	LP
Eye Removed	Enucleation	Enucleation	Phthisical eye
Preceding Surgery or Event	Repeat PKP + AMT + intravitreal Avastin	GDI	Blunt trauma
Prior Eye Surgery	PCIOL, ExPRESS, GDI	PCIOL	PCIOL
Time from Last Surgery	1 day	2.5 weeks	3 years
Topical Medications	Vancomycin (50 mg/mL) Tobramycin (14 mg/mL) Moxifloxacin Atropine	Vancomycin (50 mg/mL) Tobramycin (14 mg/mL) Prednisolone Moxifloxacin Atropine	Vancomycin (50 mg/mL) Tobramycin (14 mg/mL) Moxifloxacin Prednisolone
Periocular Injections			Subtenon's triamcinolone
Intraocular Injections	Vancomycin Ceftazidime Dexamethasone	Vancomycin Ceftazidime Dexamethasone	
Oral Medications	N/A	N/A	Levofloxacin Ciprofloxacin Prednisolone

*PCIOL* Posterior chamber intraocular lens, *n/a* Not available, *HM* Hand motion, *LP* Light perception, *NLP* No light perception, *VA* Visual acuity, *PKP* Penetrating keratoplasty, *AMT* Amniotic membrane transplantationm, *GDI* Glaucoma drainage implant

## Case reports and results

### Case 1

An 80-year-old male presented with complaints of redness and discharge two weeks following repeat penetrating keratoplasty with amniotic membrane graft and subconjunctival bevacizumab injection for corneal decompensation in the setting of multiple prior surgeries. Ocular comorbidities included a remote history of cataract surgery and ExPRESS shunt implantation, BGI for neovascular glaucoma, and a central retinal vascular occlusion. Prior to the penetrating keratoplasty surgery, baseline VA was noted to be poor (hand motion). Exam revealed purulent discharge, hypopyon, shallow anterior chamber (AC), and dense vitritis on B-scan echography. An AC paracentesis was performed for culture after a failed vitreous tap. Intravitreal vancomycin (1.0 mg/0.1 mL) and ceftazidime (2.25 mg/0.1 mL) were injected. Topical fortified tobramycin and vancomycin were initiated. VA improved to count fingers the following day, however, exam revealed possible periorbital extension with restricted ocular movement for which oral amoxicillin-clavulanate was started. The following day, further clinical decline was noted with decrease in VA to hand motion, increasing hypopyon size, and pain. Given poor visual potential and patient preference, the eye was enucleated 2 days following initial presentation.

### Case 2

A 66-year-old female presented with a painful red eye for 2 weeks. Ocular history included a prior glaucoma drainage implant (GDI) at an outside facility 6 months ago. Of note, VA prior to GDI was LP. Exam at presentation included hypopyon and fibrin surrounding the tube in the AC. B-scan ultrasound showed dense vitreous membranes. A vitreous culture was performed, and vancomycin (1.0 mg/0.1 mL), ceftazidime (2.25 mg/0.1 mL), and dexamethasone were injected. Topical fortified vancomycin 25 mg/ml, tobramycin 14 mg/ml, atropine 1% solution, and 1% prednisolone acetate were also initiated and eventually deescalated to moxifloxacin following identification of *B. cepacia.* At 8 days following initial presentation, her pain improved but visual acuity decreased to no light perception (NLP); there was a persistent hypopyon and B-scan echography demonstrated a retinal detachment. At six weeks follow-up, infection was resolved but the eye remained NLP with 360-degree choroidal and retinal detachments with phthisical changes. The eye was enucleated 3 years following initial presentation due poor prognosis and new exposure of the GDI and IOL.

### Case 3

A 62-year-old male presented with a 2-week history of progressive eye pain. The patient had prior cataract surgery 3 years earlier and reported an episode of blunt trauma (an elbow to the eye) approximately 2 weeks prior to symptom onset with stable subjective vision until the day of presentation. Presenting VA was LP and initial examination revealed complete corneal opacification with central corneal ulceration and 360 choroidal detachment on B-scan echography. The patient was taken to surgery for corneoscleral debridement of necrotic tissue and implantation of a corneal patch graft. Intraoperative biopsy was performed. The patient was treated with fortified topical vancomycin, tobramycin, with the addition of moxifloxacin upon identification of *B. cepacia*, as well as oral levofloxacin followed by oral ciprofloxacin. Once fungal infection was ruled out, topical prednisolone was initiated with subsequent addition of oral prednisolone and subtenon’s triamcinolone. Despite aggressive topical and periorbital treatment, at two-week follow-up, B-scan ultrasound showed retinal detachment, dense vitritis, and subretinal opacities suggestive of endophthalmitis. Due to poor prognosis, early phthisis bulbi, and an unsalvageable eye, a vitreous tap was deferred. Comfort measures and enucleation were recommended. The patient was subsequently lost-to-follow-up.

## Discussion

In the review of the literature, 13 additional reports of BCC related endophthalmitis were identified accounting for 56 cases (Table 3). BCC related corneal ulcers or systemic infections were not included in this review. The reports range from 1992 with Irvine and colleagues to Lind and colleagues reporting 6 cases in 2021. 78% (44/56) of cases were preceded by cataract surgery, 9% (5/56) occurred after trauma, and remainder occurring after intravitreal injections, vitrectomy, and corneal transplantation. Time to onset ranged from 2 days to 6 months from the identified inciting event, with an average of 36 days and median of 20 days. Recurrent infection was described in 13 cases, of which 6 cases had more than one recurrence. Final visual outcomes also varied significantly. One quarter of cases (14/56) had visions of 20/40 or better, with 6 of the 13 cases reported by [14]. An additional 32% (18/56) achieved a final VA between 20/40 and 20/200. The remainder had poor visual outcomes of which at least 7 ended in phthisis or enucleation and 3 had retinal detachments by the end of respective follow up periods. Recurrence was also common with reports of at least 13/56 cases.

**Table 3:** Review of literature

Study	Patient	Age	Gender	Species	Preceding Surgery/Event	Onset (days)	Recurrence	Intervention	Initial VA	Final VA	Outcome
**Irvine, 1992** [35]	1	NR	NR	*P. cepacia*	scleral laceration (trauma)	12		PPV/IOAB followed by chloramphenicol injection		20/20	Cleared infection
**Pathengay, 2005** [20]	1	53	M	*B. cepacia*	CE/IOL	30	Yes	PPV/PPL/IOAB/topicals	LP	NLP	Phthisis (LTFU after treatment of recurrence)
**Eser, 2006** [11]	1	63	M	*B cepacia*	CE/IOL	Unknown		IOAB/top, PPV/IOAB		20/63	Cleared infection, NVI
	2	72	M	*B cepacia*	CE/IOL	15		IOAB/top, PPV/IOAB	HM	20/50	Cleared infection
**Sunenshine, 2009** [31]	1	59–82	M	*B cepacia* complex	CE/IOL (contaminated trypan blue)	27		Unknown			Unknown
	2	59–82	M	*B cepacia* complex	CE/IOL (contaminated trypan blue)	94		Unknown			Unknown
	(3–6)	59–82	M	*Pseudomonas*	CE/IOL (contaminated trypan blue)	1 to 6		Unknown			Unknown
**Sachdeva, 2011** [26]	1	80	M	*B. cepacia*	CE/IOL	180		PPV/IOAB	LP	HM	Cleared infection, ERM
	2	60	M	*B. cepacia*	PK	2		PPV/IOAB	LP	20/50	Favorable, cleared infection
	3	65	F	*B. cepacia*	CE/IOL	5		PPV/PPL/IOAB	LP	CF	Retinal detachment
	4	53	M	*B. cepacia*	CE/IOL	31		PPV/PPL/IOAB	LP	NLP	Phthisis
	5	54	M	*B. cepacia*	CE/IOL/Ant vitrectomy	23		PPV/IOAB	CF	20/40	Favorable, cleared infection
	6	80	F	*B. cepacia*	CE/IOL	35		PPV/IOAB	20/200	20/80	Favorable, cleared infection
	7	7	F	*B. cepacia*	trauma	14		PPV/PPL/IOAB	NLP	NLP	Evisceration
	8	45	M	*B. cepacia*	CE/IOL	13		PPV/IOAB	CF	CF	Cleared infection, CNVM
	9	80	M	*B. cepacia*	CE/IOL	150		PPV/IOAB	LP	20/100	Favorable, cleared infection
	10	60	F	*B. cepacia*	CE/IOL	10		PPV/IOAB	LP	20/160	Favorable, cleared infection
	11	65	M	*B. cepacia*	CE/IOL	10		PPV/IOAB	LP	LP	Retinal detachment
	12	33	M	*B. cepacia*	trauma	2		PPV/PPL/IOAB removal	20/60	LP	Retinal detachment
	13	27	F	*B. cepacia*	trauma	2		PPV/PPL/IOAB removal	LP	20/20	Favorable, cleared infection
	14	35	M	*B. cepacia*	trauma	3		PPV/PPL/IOAB removal	LP	NLP	Phthisis
**Saffra, 2014** [27]	1	91	F	*B. cepacia*	IV ranizumab	11		PPV/IOAB, then ceftazidime	20/60	20/30	Cleared infection, CNVM
**Lalitha, 2014** [12]	1	69	F	*B. cepacia*	CE/IOL	15		PPV/IOAB/SOI	HM	20/200	Cleared infection
	2	46	F	*B. cepacia*	CE/IOL	13		PPV/OAB/SOI	20/80	20/200	Cleared infection
	3	64	F	*B. cepacia*	CE/IOL	42		PPV/IOAB/SOI	CF	CF	Cleared infection, persistent corneal infiltrate
	4	57	M	*B. cepacia*	CE/IOL	35		PPV/IOAB/SOI	HM	LP	Tunnel infiltrate, failed patch graft
	5	61	M	*B. cepacia*	CE/IOL	16		PPV/IOAB/SOI/EL	6/18	LP	Corneal infiltrate, edema, and stromal abscess
	6	66	M	*B. cepacia*	CE/IOL	23		PPV/IOAB/SOI	CF	LP	Cleared infection
	7	43	M	*B. cepacia*	CE/IOL	33		PPV/IOAB/SOI	20/200	20/20	Cleared infection, corneal infiltrate
	8	58	F	*B. cepacia*	CE/IOL	80		PPV/IOAB/SOI	20/80	20/80	Cleared infection, TPK
	9	70	F	*B. cepacia*	CE/IOL	73		PPV/IOAB/SOI	HM	20/60	Cleared infection
	10	61	M	*B. cepacia*	CE/IOL	45		PPV/IOAB/SOI	20/60	20/80	Cleared infection, graft clear
	11	61	M	*B. cepacia*	CE/IOL	89		PPV/IOAB/SOI	CF	20/60	Cleared infection, graft clear
	12	65	M	*B. cepacia*	CE/IOL	46		PPV/IOAB/SOI	HM	20/60	Cleared infection, persistent tunnel infiltrate
	13	49	F	*B. cepacia*	CE/IOL	92		PPV/IOAB/SOI	CF	20/40	Cleared infection, secondary glaucoma
**Deka, 2018** [6]	1	68	M	*B. cepacia*	CE/IOL	14		topical moxifloxacin/prednisone/atropine then 3d IAOB, 7d PPV/IOAB	Unknown	NLP	Phthisis
	2	59	F	*B. cepacia*	CE/IOL	21	Yes	IOAB, then PPV/IOAB	20/200	NLP	Evisceration
	3	69	M	*B. cepacia*	CE/IOL	18		IOAB	CF	20/30	Cleared infection
**Okonkwo, 2018** [17]	1	34	M	*B cepacia*	RD d/t PVR then PPV/SB/SOI	7	Yes	CE/SOR/PPV/IOAB	CF	NLP	Phthisis
	2	43	M	*B cepacia*	RD d/t PVR then PPV/SB/SOI	7	Yes	PPV/SOR/PPV/IOAB	20/120	HM	PVR with hypotony
**Okonkwo, 2020** [18]	1	34	F	*B. cepacia*	IV bevacizumab	4	Yes	IOAB/top, PPV/IOAB/SOI, PPV/PPL/IOAB	CF	20/60	Favorable, cleared infection
**Chen, 2021 **[4]	1	66	F	*B cepacia*	none	Unknown		PPV/PPL/EL/SOI/IOAB	HM	20/40	Cleared infection
**Deb, 2021** [7]	1	69	F	*B cepacia* complex	CE/IOL	3	Yes	IOAB then PPV/IOAB	CF	20/120	Cleared infection, CME + NVI
	2	58	M	*B cepacia* complex	CE/IOL	8		IOAB then PPV/IOAB	20/60	20/60	Cleared infection
	3	69	M	*B cepacia* complex	PKP	20		IOAB × 2	LP	LP	Phthisis
	4	55	F	*B cepacia* complex	CE/IOL	22	Yes	IOAB then PPV/IOAB	20/120	20/60	Cleared infection, secondary glaucoma
	5	60	M	*B cepacia* complex	CE/IOL	17	Yes	IOAB then PPV/IOAB	CF	20/200	Cleared infection, NVI
**Lind, 2021** [14]	1	73	M	*B. contaminans*	CE/IOL	13	Yes	PPV/PPL/IOAB/POAb		20/15	Cleared infection
	2	89	M	*B. contaminans*	CE/IOL	12	Yes	PPV/PPL/IOAB/POAb		20/22	Cleared infection
	3	74	M	*B. contaminans*	CE/IOL	15	Yes	PPV/IOAB/POAb		20/20	Cleared infection
	4	86	M	*B. contaminans*	CE/IOL	16	Yes	PPV/PPL/IOAB/POAb		20/20	Cleared infection
	5	59	F	*B. contaminans*	CE/IOL	36		PPV/PPL/IOAB/POAb		20/25	Cleared infection
	6	72	M	*B. contaminans*	CE/IOL	112	Yes	PPV/PPL/IOAB/POAb		20/25	Cleared infection

*F* Female, *M* Male, *CE/IOL* Cataract extraction and intraocular lens placement, *PPV* Pars plana vitrectomy, *PPL* Pars plana lensectomy, *IOAB* Intraocular antibiotic injection, *SOI* Silicone oil infusion, *SOR* Silicone oil removal, *POAb Per os* antibiotics, *RD* Retinal detachment, *CNVM* Choroidal neovascular membrane, *ERM* Epiretinal membrane, *HM* Hand motion, *CF* Count fingers, *LP* Light perception, *NLP* No light perception, *PKP* Penetrating keratoplasty

Among the 10 reports that described antibiotic susceptibilities, each tested and reported different antibiotic sensitivity panels (Table 4). Treatment strategies generally followed EVS guidelines with intravitreal vancomycin (1 mg/0.1 mL), ceftazidime (2.25 mg/0.1 mL). Vitrectomy with silicone oil was also a common therapy when presenting VA was LP or worse. Of the antibiotics tested in at least 3 reports, there was evidence of at least one BCC strain with resistance reported by one of the remaining 8 studies analyzed. Both [14] and Lalitha et al. reported strains sensitive to piperacillin/tazobactam. Case 3 reported in our cohort, however, displayed resistance to piperacillin/tazobactam. Ceftazidime was the most tested, utilized, and most commonly sensitive antibiotic at 68% (30 cases were sensitive, 16 were resistant, 2 were reported as “moderate” sensitivity, 1 was not reported). Of note, Deb et al. reported 5 cases initially treated with vitreous tap and injection of vancomycin plus ceftazidime ultimately requiring additional pars plana vitrectomy and injection of meropenem due to inadequate initial response yet reported sensitivity to ceftazidime in all isolates [7]. Fluoroquinolone sensitivities were reported by 9/10 studies, with ciprofloxacin being the most commonly tested showing 53% sensitivity among cases tested (21/40). Aminoglycosides were also commonly tested, gentamicin showing 33% sensitivity (13/40) and amikacin showing 20% sensitivity (8/41).

**Table 4:** Antibiotic susceptibilities among published reports

		Amino-glycosides			Fluoroquinolones							Cephalosporins		
Study	Patients	Gentamycin	Amikacin	Tobramycin	Ciprofloxacin	Levofloxacin	Ofloxacin	Gatifloxacin	Moxifloxacin	Minocycline	Chloramphenicol	Ceftazidime	Cefazolin	Cefotaxime
Irvine, 1992 [35]	1	0/1	0/1		0/1							1/1		
Pathengay, 2005 [20]	1	0/1	0/1		1/1						0/1	1/1		
Eser 2006 [11]	2	0/2	0/2	0/2	2/2		2/2					2/2		
Sachdeva, 2011 [26]	14	6/14	7/14		11/14						9/14	11/14	1/14	
Lalitha, 2014^12^	13	0/13	0/13	0/13	0/13	0/13	0/13	0/13	0/13		0/13	0/13		13/13
Saffra, 2014 [27]	1		0/1			1/1						1/1	0/1	0/1
Deka, 2018 [6]	3								0/2			2/2*		
Okonkwo, 2018 [17]	2	0/2	0/2		0/2							2/2**		
Lind, 2021 [14]	6	6/6	0/6	0/6	6/6							6/6		0/6
Deb, 2021 [7]	5											5/5**		
Current study	3	1/1	1/1	1/1	1/1	1/1				2/2		3/3		
Total	51	**13/40**	**8/41**	**1/22**	**21/40**	**2/15**	2/13	0/13	0/13	2/2	**9/28**	**34/50**	1/15	**13/20**
Percentage		**0.33**	**0.20**	**0.05**	**0.53**	**0.13**	0.15	0	0	1	**0.32**	**0.68**	0.07	**0.65**

	Cephalosporins					Extended Spectrum Pencillins & Carbopenams								
Study	Cefuroxime	Cefixime	Ceftriaxone	Cefoxitin	Cefepime	Ampicillin	Pipercillin-Tazobactam	Avibactam-ceftazidim	Ceftolozane-tazobactam	Imipenem	Meropenem	Vancomycin	Trimethoprim-sulfametoxazole	Nitrofurantoin
Irvine, 1992 [35]												0/1		
Pathengay, 2005 [19[]												0/1		
Eser 2006 [11]			0/2	0/2										
Sachdeva, 2011 [26]												0/14		
Lalitha, 2014 [12]	0/13						13/13							
Saffra, 2014 [27]						0/1								0/1
Deka, 2018 [6]		0/2												
Okonkwo, 2018 [17]													2/2**	
Lind, 2021 [14]	0/6						6/6	0/6	0/6	0/6	0/6		0/6	
Deb, 2021 [7]											5/5**		5/5**	
Current study					1/1		1/1				3/3		1/3	
Total	0/19	0/2	0/2	0/2	1/1	0/1	**20/20**	0/6	0/6	0/6	**8/14**	0/16	**8/16**	0/1
Percentage	0	0	0	0	1	0	**1**	0	0	0	**0.57**	0	**0.5**	0

Antibiotic susceptibilities as reported by previous published reports. *Deka et al. reported intermediate susceptibility to ceftazidime among both isolates. **Okonkwo et al. 2019 and Deb et al. 2021, only specified susceptible antibiotics though more agents were tested which were not reported

Reported cases of *BCC* endophthalmitis are rare. The first case of *B. cepacia* endophthalmitis was reported by Del Piero et al. in 1985 following trabeculectomy and extracapsular cataract extraction [8]. In the largest case series to date, Sachdeva et al. reported 14 cases of *BCC* endophthalmitis out of 744 cases in the LV Prasad Endophthalmitis registry between 2003 – 2008: 9 cases occurred after cataract surgery, 1 following PKP, and 4 in the setting of trauma [26]. In a recent literature review, [16] found 34 cases of post-surgical, culture-confirmed endophthalmitis between 1992–2018 among 8 reports, of which 85% occurred following cataract surgery and the remainder occurring after PKP, intravitreal injections [18, 27], and vitrectomies [16]. *BCC* isolates often display significant antibiotic resistance profiles in vitro [35]. A recent report of the molecular analysis of *B. contaminans* isolate from a polymicrobial corneal ulcer showed inherent resistance to fluoroquinolones, cephalosporins, carbapenems, monobactam, aminoglycosides, and sulfonamides while susceptible to tetracylclines, meropenem, and ceftazidime [13].

Outcomes for BCC endophthalmitis varied significantly across the 13 reports reviewed. Clear trends remain difficult to extrapolate as context and treatment strategies varied widely. Additionally, the wide degree of antibiotic resistance variation among the BCC strains, discussed further below, requires an individualized approach to care. Indeed, recurrence was noted in 13 cases, 6 of which had multiple episodes, while 5 cases reported by Deb et al. treated with an antibiotic subsequently noted as sensitive had an inadequate response.

Review of the susceptibilities of BCC related endophthalmitis cases published in the literature demonstrates a diversity of antibiotic sensitivities among the virulent strains described as well as diverse panels of antibiotic susceptibilities tested. Ceftazidime was the most commonly tested and most commonly sensitive antibiotic, followed by ciprofloxacin and gentamicin. Pipercillin/tazobactam stands out as sensitive in 19/19 cases. However, this represents only two reports [14, 15] which described two single outbreak clusters respectively, and case #3 described in the present report displayed piperacillin/tazobactam resistance. These results align well with the various resistance mechanisms employed by this bacterial genus. Given the broad resistance patterns seen, identification of BCC as well *Pseudomonas*, which is often initially misidentified for BCC, may benefit from a broader susceptibility panel examination and lower threshold to broaden therapeutic choice of agents.

In the current study, all three isolates were susceptible to ceftazidime (Table 1) which were given as intravitreal injections to patient 1 and patient 2. All patients received topical fortified vancomycin and tobramycin. In addition, all three patients received topical moxifloxacin upon identification of the causative organism. Despite *in* vitro susceptibility testing, multiple clinical and laboratory studies have substantiated the observed treatment failure seen in many patients. Although some have attributed this to inadequate antibiotic concentrations, factors like broad intrinsic antibiotic resistance, [36] biofilm formation [9], and phase of growth [21] affect the resistance profile. Various studies have shown potential BCC resistance to all the major antibiotic classes, including polymyxins, aminoglycosides [5], ß-lactams [22], fluoroquinolones [36] and tetracyclines [3]. These factors might support an earlier surgical intervention with vitrectomy [25].

All 3 patients had previous intraocular surgery. Cataract surgery is the most common cited preceding factor to *BCC* endophthalmitis [19, 26]. Time to presentation is also quite variable, as seen among our 3 cases [12, 26]. Although cases of *BCC* endophthalmitis generally meet the European Society of Cataract & Refractive Surgeons (ESCRS) criteria for acute post-operative endophthalmitis of within 6 weeks of surgery, it is notable that only 14–22% of acute endophthalmitis is seen beyond 2 weeks of surgery while *BCC* on average presents 2–6 weeks after initial insult [2]. And while prior studies comparing outcomes of acute vs delayed onset endophthalmitis found better outcomes with the more insidious forms, [29] delayed onset cases of BCC do not follow this general trend [12, 14, 26, 31]. A recent outbreak of endophthalmitis at a surgical center in Norway included 6 patients in the period from 1/4/2019 to 1/28/2019 with *B. cepacia* complex linked to a contaminated fresh air ventilation system [14, 30]. All 6 eyes had severe vision loss and required prolonged treatment course. In sharp contrast with other prior reports, Lind and colleagues reported good visual outcomes (final vision of 20/25 or better) among 6 cases of *BCC* endophthalmitis following cataract surgery treated with vitrectomy, lensectomy with complete removal of the capsular bag, and repeated intravitreal antibiotic injections [14]. These cases along with a report by Okonkwo [18] suggest an important role in capsular bag removal during initial vitrectomy as means of preventing recurrence which has been documented in other prior reports.

In the non-ophthalmic literature, *B. cepacia* infection has also been associated with immunocompromise, particularly in patients with cystic fibrosis and chronic granulomatous disease (CGD) [21]. None of our patients had a history of cystic fibrosis, CGD or known immunocompromise. Several case series have also found contaminated surgical or pharmacologic agents as sources of cluster infections which was not suspected as the likely cause in our case series [12, 31].

The current study adds 3 cases *B. cepacia* endophthalmitis, a rare clinical entity, to the body of literature. Our review of antibiotic sensitivity profiles among BCC endophthalmitis reports is the largest review to date and builds on prior reports of broad antibiotic resistances seen in BCC.

## Data Availability

The datasets used and/or analyzed during the current study are included in this published article and are also available from the corresponding author on reasonable request.

## Abbreviations

AC: Anterior chamber
AMT: Amniotic membrane transplantation
BCC: *Burkholderia* *cepacia* Complex
CF: Count fingers
CE/IOL: Cataract extraction/intraocular lens placement
CGD: Chronic granulomatous disease
CNVM: Choroidal neovascular membrane
ERM: Epiretinal membrane
EVS: Early Vitrectomy Study
F: Female
GDI: Glaucoma drainage implant
HM: Hand motion
IOL: Intraocular lens
LP: Light perception
M: Male
N/A: Not available
NLP: No light perception
NT: Not tested
NVI: Neovascularization of the iris
PCIOL: Posterior chamber intraocular lens
PKP: Penetrating keratoplasty
POAb: *per **os* Antibiotics
PPL: Pars plana lensectomy
PPV: Pars plana vitrectomy
RD: Retinal detachment
VA: Visual acuity
